# Case report of metastatic Merkel cell carcinoma presenting as purpuric chest discoloration

**DOI:** 10.1016/j.jdcr.2026.04.001

**Published:** 2026-04-09

**Authors:** Julianna Glienke, Jennifer Burris, William Rietkerk

**Affiliations:** Conejo Dermatology, Thousand Oaks, California

**Keywords:** localized erythematous, Merkel cell carcinoma, metastatic, purpura

## Introduction

Merkel cell carcinoma (MCC) is a rare neuroendocrine tumor located in the epidermis, characterized by rapid growth and metastasis.^1^^,^^2^ The typical initial clinical presentation of an MCC is a firm, asymptomatic nodule, often exhibiting a shiny appearance.^1^^,^^2^ Metastatic MCC typically presents as satellite lesions similar to the initial presentation. Here, we report the metastasis of MCC with a novel clinical presentation of an erythematous to purpuric patch on the chest.

## Case report

The patient was a 93-year-old Caucasian male with a history of metastatic castrate-resistant prostate cancer (mCRPC) and recurrent MCC. The patient was initially diagnosed with stage III-A MCC in June 2020, identified by a shave biopsy on the right forearm. A new primary MCC was diagnosed in the left submandibular area in June 2023 as a small pink to skin-colored nodule and was subsequently confirmed with a biopsy. With evidence of early MCC recurrence in 2024, the patient was under the care of a hematology oncology specialist for clinical progression of submandibular mass on the left face and an oncologist for treatment of prostate cancer with metastasis to the bone. In 2024, the patient started cabazitaxel for prostate cancer and pembrolizumab for the local advancement of the MCC. In March 2025, the patient had a purpuric-to-erythematous patch on the sternum that was initially thought to be allergic contact dermatitis with associated purpura due to concurrent use of blood thinners. He was asymptomatic for the discoloration.

**Fig 1:**
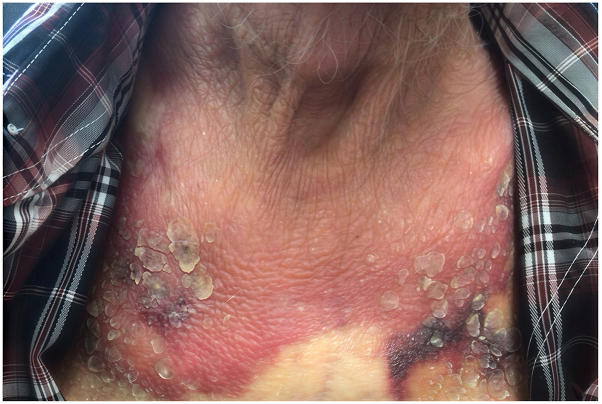
Clinical presentation of 10% BSA localized discoloration on the chest. *BSA*, Body surface area.

**Fig 2:**
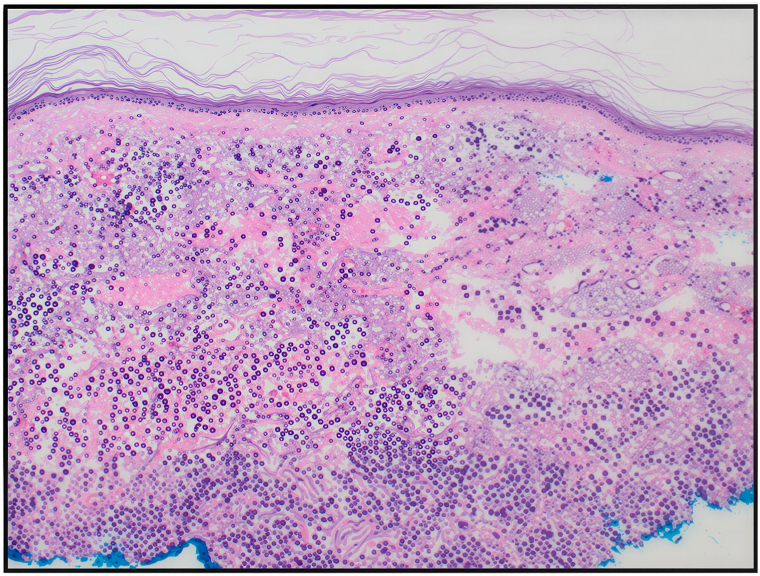
Cells arranged in nests and sheets in the superficial dermis.

The patient was prescribed topical steroids (triamcinolone acetonide 0.025% topical cream) to minimize any itching or irritation. During the subsequent visit in May, he had worsening of the discoloration, and the purpura had mostly resolved, leaving an erythematous to slightly violaceous patch on the upper chest (Fig 1). Due to the patient’s previous history and failure to improve with topical steroids, a shave biopsy was performed on the right clavicular skin. The specimen was sent to a dermatopathologist for histopathologic evaluation. The histology revealed a population of rounded cells arranged in nests and sheets in the superficial dermis (Fig 2).

**Fig 3:**
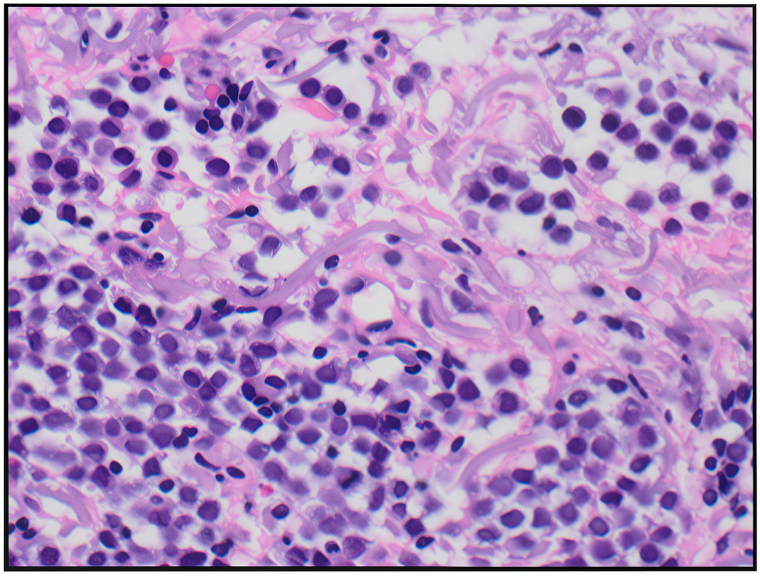
Small blue round-to-oval cells proliferation shown with scant cytoplasm and enlarged hyperchromatic nuclei.

The lesional cells had enlarged hyperchromatic nuclei and scant cytoplasm (Fig 3).

**Fig 4:**
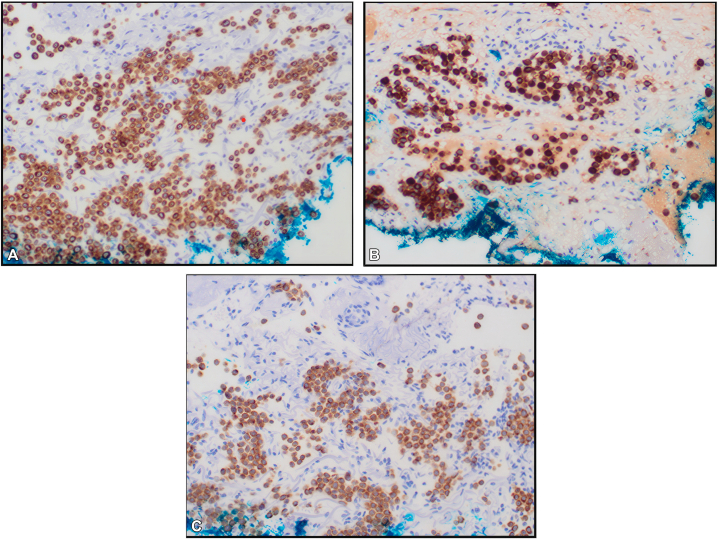
**A,** CK20 stain demonstrated brown stained dot-like paranuclear staining from clumping of intermediate filaments. **B,** Chromogranin stain revealed cells positive for chromogranin A within the cytoplasm of tumor cells. **C,** Cytoplasmic staining with synaptophysin proteins found in the presynaptic vesicles of neurons and neuroendocrine cells.

Immunohistology showed positive expression of cytokeratin 20, synaptophysin, and chromogranin, characterizing the lesional cells as neuroendocrine and epithelial markers (Fig 4, *A*-*C*).

The lesional cells were negative for prostate-specific antigen, cluster of differentiation 3, cluster of differentiation 20, and cytokeratin 7. The tumor had a thickness of 0.7 mm, with lymphovascular invasion, and a mitotic rate of 3 per high-power field. The tumor-infiltrating lymphocytes were present and nonbrisk. These findings confirmed the diagnosis of an MCC, and the patient was referred to their current oncologist for the treatment of metastatic MCC. The patient was also referred to a radiation oncologist for further treatment options. After a discussion of treatment options from both the oncologist and radiation oncologist for the treatment of metastatic MCC and metastatic prostate cancer, the patient decided to stop all cancer-directed treatment.

## Discussion

Primary MCC typically presents as a painless, firm, skin-colored to purple-red nodule, often on sun-exposed areas.^2^ Recurrence and metastasis to nearby areas can be diagnosed with preoperative and follow-up positron emission tomography/computed tomography scans. Metastasis most commonly occurs in distant lymph nodes, liver, and lungs.^3^ Satellite lesions can occur as MCC extends into the subcutaneous tissue and travels locally via dermal lymphatics.^4^ Satellite tumors usually present as small, new nodules adjacent to the primary tumor.^5^ While localized discoloration isn’t typically present in the clinical presentation of MCC, erythematous plaque and possible bruising related to a primary nodular lesion have been documented before.^6^ However, in this case, there was a novel clinical presentation of purpuric discoloration not directly adjacent to the primary tumor, representing metastatic growth. The presence of a large erythematous patch on the anterior neck could demonstrate distinct lymphatic spread from the primary tumor. Additionally, a complicating factor for this patient was the presence of 2 separate metastatic carcinomas and how the treatment for these cancers could have interacted. There is very little research on the effect of combining cabazitaxel and pembrolizumab for the treatment of MCC. The combination of chemotherapy and immunotherapy could lead to cabazitaxel counteracting pembrolizumab’s efficacy for controlling MCC’s progression. This combination was necessary because pembrolizumab for MCC is the most effective immunotherapy treatment, while cabazitaxel minimizes the progression of the patient’s prostate cancer. Cabazitaxel, while targeting cancer cells, can specifically damage T cells, which are crucial for pembrolizumab effectiveness. This can reduce the pembrolizumab’s ability to stimulate an effective antitumor immune response to slow the patient’s MCC effectively. Chemotherapy would be less feasible for an elderly patient with comorbidities and a weakened immune system, and MCC tends to acquire quick resistance to most chemotherapy treatments. A current clinical trial is underway to investigate the synergistic effect of cisplatin in combination with pembrolizumab.^7^ If the trial results are positive, this could be an option for treating both MCC and mCRPC, since platinum-based chemotherapy and pembrolizumab are treatment options for mCRPC. In summary, this patient’s case presents a novel clinical presentation of metastatic MCC as an erythematous patch without satellite lesions. We can additionally consider that the patient had 2 aggressive cancers requiring different courses of treatment, and these treatments could affect the overall treatment efficacy. Further research could investigate the effects of cabazitaxel and pembrolizumab that could have negatively impacted the patient.

## Conflicts of interest

None disclosed.
